# Timing of anticoagulation for the management of portal vein thrombosis in liver cirrhosis

**DOI:** 10.2478/jtim-2023-0083

**Published:** 2023-07-05

**Authors:** Emanuele Valeriani, Pasquale Pignatelli, Marco Senzolo, Walter Ageno

**Affiliations:** Department of General Surgery and Surgical Specialties, Sapienza University of Rome, Rome 00185, Italy; Department of Infectious Disease, Azienda Ospedaliero-Universitaria Policlinico Umberto I, Roma 00161, Italy; Department of Clinical, Internal, Anesthesiological and Cardiovascular Sciences, Sapienza University of Rome, Rome 00185, Italy; Multivisceral Transplant Unit-Gastroenterology, Azienda Ospedaliera Universitaria di Padova, Padova 35128, Italy; Department of Medicine and Surgery, University of Insubria, Varese 21100, Italy

## Introduction

Portal vein thrombosis (PVT) represents a not uncommon complication of liver cirrhosis with an impact on patients’ morbidity and, possibly, patients’ mortality.^[1–3]^ The prevalence of PVT is associated to the severity of liver disease and ranges from about 1% in patients with compensated liver cirrhosis to 8% in patients with decompensated liver cirrhosis and up to 40% in patients who developed a hepatocellular carcinoma.^[2,4]^ Despite recent evidence, thrombus localization and extension as well as the perceived increase in bleeding risk due to both hemostatic abnormalities (*e.g*., defective platelet number or function) and portal hypertension (*e.g*., high-risk gastroesophageal varices) may complicate the decision of which patients should be treated and when anticoagulant treatment should be started.^[3,5,6]^

A recent guidance paper from the International Society of Thrombosis and Haemostasis suggested that the stage of thrombosis (*i.e*., acute or chronic) rather than the presence or absence of symptoms at diagnosis should guide therapeutic decision.^[5]^ Anticoagulant therapy is suggested for patients with acute symptomatic or incidentally-detected PVT without active bleeding or other contraindications.^[5]^ Conversely, a careful evaluation of the risks and benefits of anticoagulation is suggested in patients with chronic thrombosis.^[5]^ The aims of this narrative review are to discuss the available data and to identify the correct timing for anticoagulant therapy in patients with liver cirrhosis and PVT.

## Anticoagulant Therapy in Acute or Recent Pvt

Acute or recent PVT is less common than a chronic thrombosis in cirrhotic patients but may cause an increase in portal pressure and an higher risk of portal hypertension-related bleeding.^[7]^ The presence of preexistent portosystemic collaterals of liver cirrhosis may partly reduce the risk or the severity of these complications.^[8]^ However, the main scopes of anticoagulant therapy in acute PVT are to achieve vessel recanalization, to reduce the risk of both ischemic and bleeding complications throughout thrombus resolution, and to allow physiological portal vein inflow at transplantation.^[9, 10, 11]^ Data from recent meta-analysis confirmed the efficacy of anticoagulant therapy in liver cirrhosis-related PVT, with significantly increased rates of vein recanalization (relative risk [RR], 3.19; 95% confidence interval [CI], 1.42 to 7.17) and significantly lower rates of thrombus progression (RR, 0.28; 95% CI, 0.15 to 0.52) as compared to untreated patients, and a significant reduction of both major bleeding (RR, 0.52; 95% CI, 0.28 to 0.97) and gastroesophageal variceal bleeding risks (RR, 0.26; 95% CI, 0.11 to 0.65).^[12,13]^ These results were also observed in a large prospective cohort of cirrhotic patients with splanchnic vein thrombosis, with significantly higher bleeding rates in never treated patients as compared to treated patients, probably due to PVT-related hemodynamics.^[1]^ If the need for anticoagulant treatment in patients with cirrhosis-associated PVT is supported by increasing evidence, the optimal timing for starting treatment remains controversial, evidence is lacking, and individual patient’s characteristics solely drive the decision.^[5]^ A small retrospective study including 66 patients with liver cirrhosis-related PVT reported that the time between diagnosis and starting of treatment was not associated with vein recanalization. However, no information was provided about the stage (*i.e*., acute or chronic) of thrombosis and about half of anticoagulated patients stopped treatment prematurely, mostly due to patients’ decision or minor bleedings.^[14]^ Another retrospective study including 55 cirrhotic patients with acute or recurrent PVT reported that anticoagulant treatment started within 14 days from diagnosis was associated with higher rates of vein recanalization than a delayed treatment strategy.^[15]^ Data on a longer time interval between PVT diagnosis and treatment administration (*i.e*., 3 to 6 months) were conflicting possibly due to the different methodology for the estimation of thrombosis occurrence time.^[16, 17, 18]^ For example, the presence of symptoms, the analysis of previous radiological imaging, and the radiological characteristics of the thrombus at diagnosis were taken into account in two studies, while any information regarding the methodology to assess the stage of thrombosis were reported in a latter study.^[16, 17, 18]^ Consequently, it should be acknowledged that the proportion of patients with acute/recent or chronic PVT differed across these studies.^[16, 17, 18]^ This lack of knowledge hampers clinical decision that remains guided by experts’ opinion and mainly based on patients’ characteristics and prognosis.

The presence of esophageal varices may pose some concerns about the safety of a prompt starting of anticoagulant treatment, early esophagogastroduodenoscopy is not always feasible, and anticoagulant therapy may complicate this latter procedure.^[19]^ However, the presence of esophageal varices should not represent an absolute contraindication to anticoagulant therapy as most of patients with PVT and liver cirrhosis are already receiving prophylaxis.^[8,20]^ Data from a recent retrospective study comparing the outcomes of varices endoscopic treatment in PVT patients receiving low molecular weight heparin and in patients receiving no therapy showed that anticoagulation did not increase the risk of both post-procedural bleeding (3.8% *vs*. 1.6%; hazard ratio 1.7, 95% CI 0.2 to 21.2) and mortality (2.5% *vs*. 2.2%; hazard ratio 0.9, 95% CI 0.1 to 16.6). Of note, 65% of treated patients were receiving therapeutic dose anticoagulation and 95% had high risk varices.^[21]^ Rates of post-procedural variceal bleedings (9%) were higher in a more recent retrospective study, but about one third of patients were on vitamin K antagonists and no information was provided on drugs and dosages associated with bleeding events.^[22]^

**Figure 1:**
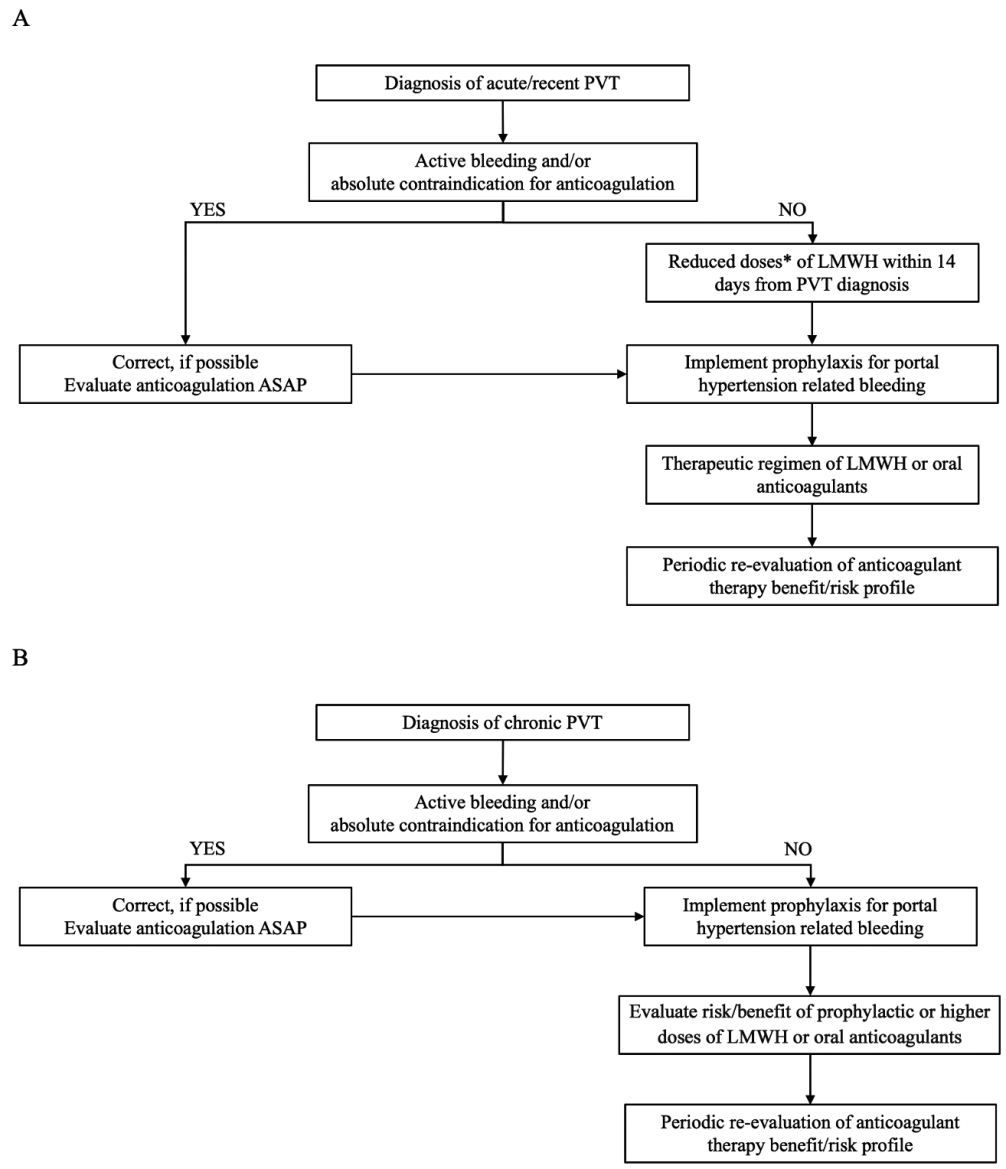
Therapeutic management of patients with acute (A) and chronic (B) portal vein thrombosis. *Prophylactic or intermediate doses of LMWH. ASAP, as soon as possible; LMWH, low molecular weight heparin; PVT, portal vein thrombosis.

Based on this limited evidence, experts have suggested to start treatment with a reduced dose of low molecular weight heparin (*i.e*., prophylactic or half therapeutic dose) within 14 days from PVT diagnosis in the absence of active bleeding or other contraindications.^[20]^ The dose of low molecular weight heparin may be increased to therapeutic doses, or oral anticoagulant therapy may be started after a proper endoscopic and/or pharmacological prophylaxis for esophageal varices has been achieved.^[20]^ Figure 1A reports the proposed therapeutic approach for patients with liver cirrhosis and acute PVT.

## Anticoagulant Therapy in Chronic Pvt

Chronic PVT is generally characterized by signs of longstanding thrombosis including the presence of intra-abdominal venous collaterals or cavernous transformation of the portal vein.^[9]^ The rates of vessel recanalization achieved during anticoagulation in patients with chronic PVT are lower than in patients with acute PVT.^[5]^ The main goal of anticoagulant therapy in patients with chronic thrombosis is to prevent recurrent events and thrombosis progression that may worsen splanchnic hemodynamics.^[9]^ Furthermore, the presence of PVT has a significant impact on transplant and post-transplant outcomes. The decision to start anticoagulant therapy should, therefore, be considered in selected patients only, in whom the benefit of preventing extension of thrombosis is greater than the estimated risk of bleeding. This decision should also consider patient preferences, the effects on quality of life, and costs.^[5]^ In a prospective cohort study, prophylactic dose of low molecular weight heparin was associated with lower rates of thrombosis progression in patients with chronic PVT compared with no treatment (14.3% *vs*. 71.4%).^[18]^ The need and dose of anticoagulant therapy should be periodically reassessed to ensure a favorable risk to benefit profile.^[5]^ Figure 1B reports the proposed therapeutic approach for patients with chronic PVT.

## Conclusion

Anticoagulant therapy provides beneficial effects in portal hemodynamics of patients with both acute and chronic PVT. A prompt start of therapy should be considered for most patients, specifically in whom thrombotic risk exceeds the bleeding risk. In patients with high-risk varices, adequate pharmacological and/or endoscopic prophylactic strategies should be implemented as soon as possible. When indicated, anticoagulation should be started within the first 2 weeks from the diagnosis of acute PVT, possibly starting with reduced doses of low molecular weight heparin that may be increased or switched to oral anticoagulation based on patients and thrombosis characteristics.
